# SKS-transformer: multi-scale and direction-aware attention for inertial sensor-based activity recognition

**DOI:** 10.3389/fspor.2026.1754717

**Published:** 2026-01-23

**Authors:** Chengwei Feng, Boris Bačić, Weihua Li, Hongqi Xu

**Affiliations:** 1 School of Engineering, Computer and Mathematical Sciences (ECMS), Auckland University of Technology, Auckland, New Zealand; 2 Department of Data Science and Artificial Intelligence, Auckland University of Technology, Auckland, New Zealand; 3 Institute of Biomedical Technologies (IBTec), Auckland University of Technology, Auckland, New Zealand; 4 Sports Performance Research Institute New Zealand (SPRINZ), Auckland University of Technology, Auckland, New Zealand; 5 Research Center of Sports and Health Science, School of Sports Science and Physical Education, Northeast Normal University, Changchun, Jilin, China

**Keywords:** biomechanics, Human Activity Recognition (HAR), inertial data classification, IoT, open source (OS), privacy & security, transformer, wearable IMU sensors

## Abstract

**Introduction:**

Human Activity Recognition (HAR) has emerged as an enabling research field, with applications ranging from healthcare and sports analytics to smart environments. However, achieving scalable and accurate HAR systems that generalize across diverse activity scenarios remains a challenging problem.

**Methods:**

In this paper, we propose a scalable HAR system, which integrates a new model named SKS-Transformer with a custom-designed wearable Inertial Measurement Unit (IMU). The IMU combines an ESP8266 microcontroller and a JY61 sensor, enabling wireless acquisition of motion data. The proposed SKS-Transformer model incorporates Selective Kernel Networks and squeeze-enhanced axial attention modules to capture multiscale temporal dynamics and directional dependencies, respectively. The motion data preprocessing pipeline includes denoising, segmentation, and normalization. The preprocessed data are fused through a learnable gating mechanism, enabling the model to adaptively balance local and global motion patterns.

**Results:**

We evaluate the system scalability and performance on two public datasets (UCI-HAR and PAMAP2) and two captured datasets that feature both daily activities and fine-grained golf swing errors. Experimental results demonstrate that the SKS-Transformer model consistently surpasses the state of the art on both public datasets (by 0.3% and 0.09% compared to the best of 11 other published models) and by 2.86% and 0.46%, achieving the accuracy of up to 98.10% on collected HAR data, as well as 100% accuracy in golf swing error detection.

**Discussion:**

Ablation studies of SKS-Transformer confirm the contribution of each architectural model component to overall model performance and provide further insights for future optimizations. Future work will investigate the applications of the SKS-Transformer-based system in extended real-world scenarios, including intelligent healthcare, sports performance monitoring, and wearable computing. The source code for our proposed method has been released publicly and is available on GitHub at: URL: https://github.com/cw-feng/SKS-Transformer-Multi-scale-and-direction-aware-attention-for-activity-recognition.

## 1 Introduction

Human Activity Recognition (HAR) plays a pivotal role in a wide range of real-world applications, such as health and activity monitoring, elderly care, rehabilitation, sports training, and human-computer interaction (1–4). With the proliferation of wearable devices, smartphone sensors, and the rapid advancement in edge computing and machine learning, inertial sensor-based HAR systems have gained significant attention due to their low power consumption, portability, and resilience in unconstrained environments compared to Computer Vision-based (CV-based) or Radio-Frequency-based (RF-based) systems (5, 6).

Despite substantial progress in sensor-based HAR, several challenges remain. Primarily, raw data obtained from inertial measurement units (IMUs) are often noisy and complex, exhibiting strong temporal dependencies, inter-subject variability, and activity overlap. Traditional approaches, such as handcrafted features or shallow classifiers, struggle to capture these dynamics robustly. While deep learning approaches have shown potential, many depend on Convolutional Neural Networks (CNNs), which are effective at extracting local features but lack temporal memory, or on recurrent architectures (e.g., LSTM, GRU), which face limitations in parallelization and capturing long-range dependencies (7). Secondary challenges arise from the limitations of most methods, which focus on generic datasets in laboratory settings, thereby limiting their generalization to domain-specific and real-world use cases (8, 9).

Several recent works have attempted to address sensor-based HAR challenges by leveraging deep learning models, where CNNs such as AlexNet and ResNet have been widely adopted to extract local spatial features from IMU time series (10, 11). However, CNN models are primarily designed for image data and often produce redundant features when applied to motion sequences, thereby limiting their ability to capture temporal continuity. To model sequential dependencies, recurrent architectures such as LSTM networks and hybrid CNN-LSTM models have been proposed by (12, 13). Such recurrent models are effective at capturing time-dependent patterns but suffer from limitations such as vanishing gradients, limited parallelism, and increased computational overhead, particularly when applied to long-time windows or high-resolution sensor data (7). More recently, transformer-based models have gained attention in recent human activity recognition tasks for their effectiveness in modeling long-range dependencies. For example, in a systematic review by Chen et al. (14), the authors discussed trends in AI applications in sports, including the rise of transformer-based models. Originally, transformer-based models were designed for processing natural language or computer vision data and are therefore not inherently tailored to the structural characteristics of sensor signals. In particular, IMU-based motion data exhibit strong temporal locality, periodicity, and directional variation across sensor axes. Therefore, for transformer-based models that rely on global self-attention, this would limit their ability to capture spatiotemporal patterns in IMU signals effectively. Moreover, the uniform treatment of input tokens typical of common transformer-based models may dilute subtle yet meaningful motion cues across different temporal scales, which are critical for accurately recognizing fine-grained activities.

### 1.1 Research objectives

To address the sensor-based HAR challenges introduced, we propose an end-to-end human activity recognition system that integrates a custom-designed, low-cost IMU-based wearable device with a novel transformer-based model, named *SKS-Transformer*. The system design is intended to be adaptable, low-power, modular, and scalable, supporting accurate motion tracking in both generic daily activities, such as walking, sitting, or stair climbing, and fine-grained motion patterns that involve subtle biomechanical variations, such as distinguishing between correct and faulty golf swings. These fine-grained motion pattern recognition scenarios (in golf swings) require not only broad temporal modeling but also high sensitivity to localized motion cues.

As part of the intended design, we require a software pipeline that supports real-time data acquisition from IMU, preprocessing, storage, and visualization. The presented proof-of-concept software includes IMU signal denoising, sliding-window segmentation, label filtering, and normalization to prepare the input for the classifier models. The system should also operate with non-proprietary IMUs, i.e., a low-cost open-source IoT hardware (e.g., ESP8266 microcontroller with JY61 sensor), and support wireless streaming of accelerometer and gyroscope data. In addition, the end-to-end system with IoT IMU sensors should not rely on overseas cloud services.

The system should be evaluated on public benchmark datasets, such as UCI-HAR and PAMAP2, as well as custom datasets collected through the intended IoT IMU sensor platform, encompassing daily activities and fine-grained activities such as identifying golf swing errors. In summary, the key objectives of this research are:

- To introduce a real-time, low-cost, and open-source IMU-based motion data processing system, suitable for both research and real-world privacy-preserving deployment scenarios, which are not dependent on overseas cloud infrastructures.
- To extend the application scope of transformer-based AI models beyond conventional text and vision domains by adapting and validating them for effective temporal representation learning on IMU-based motion data.
- To evaluate the effectiveness of the proposed system in health activity monitoring and sports biomechanics on multiple datasets, including public and collected datasets, highlighting its potential for athletic performance monitoring and intelligent coaching.

The remainder of this paper is organized as follows. Section 2 presents methods and materials, including an IMU-based wearable system and deep learning techniques involved in the design of the proposed SKS-Transformer model. Section 3 reports results of experimental work, including performance comparison and ablation studies. Section 4 presents a broader perspective, including practical considerations and limitations, and summarizes the design aspects. Finally, Section 5 concludes the paper and outlines future research directions.

### 1.2 Background and related work

We organize the related work into three categories: (i) IoT-based wearable systems, (ii) deep learning models for sensor-based HAR, and (iii) attention-based models for time-series analysis. For ease of reading, given the broad convergence of multidisciplinary perspectives, each of the three reported categories provides a synthesis, rationale, and justification for the experimental design presented in the next sections.

#### 1.2.1 IMU-based wearable systems

IMU sensors, typically consisting of accelerometers and gyroscopes, are widely used to capture human motion by recording acceleration, angular velocity, and orientation data. Numerous studies have explored the design of wearable IMU systems for activity recognition and biomechanical analysis. For instance, in the work of (15), a wearable system dependent on IMU sensors and related algorithms was developed for human gait analysis, highlighting the potential of IMUs in clinical applications. Similarly, (16) investigated wearable sensors for detecting gait phases, demonstrating the feasibility of continuous real-time monitoring in rehabilitation scenarios. More recently, advances in low-cost hardware components, such as the ESP8266 microcontroller and MPU6050 sensor, have enabled the development of affordable, scalable IMU systems. For example, research by Landa-Jimenez et al. (17) focused on designing a low-cost IMU system using the ESP8266 microcontroller for the acquisition of human movement data. While these systems have demonstrated satisfactory performance in controlled environments, they have provided valuable insights for developing our own low-cost IMU system.

The transmission and processing of sensor data are critical components of any IMU-based system. Wireless data transmission protocols, such as Wi-Fi and Bluetooth, are often employed to enable real-time communication between wearable devices and server systems (18). Sharma et al. (19) introduced an inertial navigation system (INS) focusing on secure communication, data analysis, signal processing, and robust algorithms. Their research investigates and develops methods to address security, confidentiality, and data integrity, aiming to ensure high data quality for users and information management systems. Similarly, (18) proposed an IoT-based architecture for continuous motion monitoring, where sensor data is transmitted to a centralized server for storage and processing.

While current IMU-based systems can perform basic human motion capture tasks, they are often constrained to specific application scenarios and lack adaptability across different tasks and environments. Moreover, the closed, vendor-locking nature of common commercial solutions typically imposes limitations on data exchange and integration with broader research and development analytics environments, which would otherwise further promote the use of technology for societal benefit. To address these limitations, we aim to introduce a highly customizable, fully open-source IMU sensing platform that supports both wearable and embedded data acquisition modes. Designed for scalability and cross-system compatibility, the intended platform should support open-source software integration and a wide range of applications, from daily activity recognition to high-precision biomechanical analysis.

Beyond commercial and proprietary solutions, there is a segment of existing IMU hardware and platforms developed initially for general-purpose data acquisition or interactive demonstrations. Given the need for downstream motion-data modeling, the authors view this as a research opportunity in which data collection and model training can help identify issues such as high noise, signal drift, and inconsistent data formatting. These problems, in turn, increase the preprocessing burden and degrade the performance and generalizability of HAR models. As a rationale for our decision, our proposed platform addresses this challenge by adopting a sensor-model co-design paradigm that produces high-quality, low-redundancy, semantically structured data tailored for machine learning pipelines.

#### 1.2.2 Deep learning models for sensor-based HAR

According to (20), deep learning has become the de facto standard for HAR using time-series sensor data. In that sense, CNNs are widely used for feature extraction because they model local spatial dependencies (21, 22). However, standard CNNs lack temporal memory, rendering them suboptimal for tasks that require long-range temporal reasoning. To mitigate this, hybrid CNN-based architectures (e.g., CNN-LSTM, CNN-GRU) have been proposed that combine local feature extraction with temporal modeling (23, 24). While these architectures demonstrate improved performance, they often suffer from sequential bottlenecks, vanishing gradients, and limited parallelism during training.

Bidirectional LSTM (BiLSTM) models have also gained traction for HAR due to their ability to access both past and future contexts within a time window (25). However, they still rely on fixed-size input windows and struggle to generalize across diverse activity scales and sensor placements without tailored pre-processing.

On the other hand, transformer-based architectures, originally designed for natural language processing, have shown promising results in sensor-based HAR, where their self-attention enables modeling of long-range temporal dependencies (26). In an earlier study, (27) adapted standard transformers to sensor data by flattening input sequences or applying temporal positional encoding. Despite improved performance over RNN-based methods, generic (also called “vanilla”) transformers treat all time steps uniformly, which is suboptimal for HAR tasks where meaningful patterns often occur at specific local temporal scales or within specific sensor channels. To address this shortcoming, Pareek et al. (28), have proposed hybrid CNN-Transformer models in which convolutional layers capture local features before feeding them into the transformer model. Yet, these models often lack mechanisms to adaptively select relevant temporal scales or directional attention, limiting their expressiveness in real-world HAR settings.

All things considered, we identify the need for a mechanism or model design that can adaptively capture multi-scale temporal dynamics while disentangling temporal and feature-wise dependencies. Integrating these design requirements into a transformer-based framework is a promising research direction for efficiently modeling both local and global motion patterns.

#### 1.2.3 Multi-scale and attention mechanisms for HAR

Attention mechanisms have demonstrated strong potential in time-series modeling by allowing networks to selectively focus on temporally or spatially salient features within the data (29). In the context of human HAR, temporal attention modules have been employed to emphasize discriminative time steps within an activity sequence, thereby improving both interpretability and classification accuracy (30). On the other hand, spatial attention techniques, particularly in multichannel sensor setups, prioritize sensor modalities or body locations based on their relative contribution to specific activities (31). These methods, while effective in constrained scenarios, often struggle when faced with fine-grained, dynamic, or multiscale motion patterns, such as those encountered in rehabilitation or athletic movement analysis.

To address temporal-scale variability, (32) proposed multi-branch convolutional attention mechanisms, such as Selective Kernel Networks (SKNet). These models dynamically adjust the receptive field size to better accommodate variable-length features. However, most existing SKNet-based architectures are designed for image tasks and lack explicit temporal alignment. When applied directly to sensor data, their effectiveness is reduced by the absence of domain-specific modeling, such as directional constraints and sequential order.

Furthermore, despite the inherent directionality and axis specificity of inertial sensor data (e.g., 3D accelerometer signals), few HAR models have incorporated direction-aware attention mechanisms. Standard self-attention layers treat each time step or feature vector as a token in isolation, ignoring the geometric structure and physical interpretation of IMU channels (e.g., *x*-axis acceleration vs. *y*-axis angular velocity). This limits the model’s ability to reason over motion directionality and temporal symmetry, both of which are critical in activities involving posture changes or repetitive cycles.

To overcome these limitations, we propose a novel dual-path attention mechanism that integrates (i) Selective Kernel Networks, which extracts multi-scale temporal patterns through dynamically reweighted convolutional branches, and (ii) Squeeze-enhanced Axial Attention, which separately captures dependencies along the temporal and feature dimensions. Such a dual-path attention mechanism and decompositions enable the model to retain temporal sequencing and directional awareness without incurring the computational overhead of full self-attention. Finally, a learnable gating module adaptively fuses these two streams, allowing the network to balance global and local information pathways on a per-sample basis. To advance the state of the art, a motion data processing architecture should facilitate more robust generalization across both coarse-grained (e.g., walking vs. running) and fine-grained (e.g., correct swings vs. incorrect ones) HAR tasks, making it particularly suitable for high-resolution motion classification in unconstrained environments.

## 2 Methods and materials

The proposed HAR system integrates hardware and software components enhanced by machine learning algorithms. The system enables real-time motion data collection and autonomous operation through a novel deep learning architecture trained on wearable sensor data.

### 2.1 Overall architecture of IMU-based motion capture system

**Figure 1:**
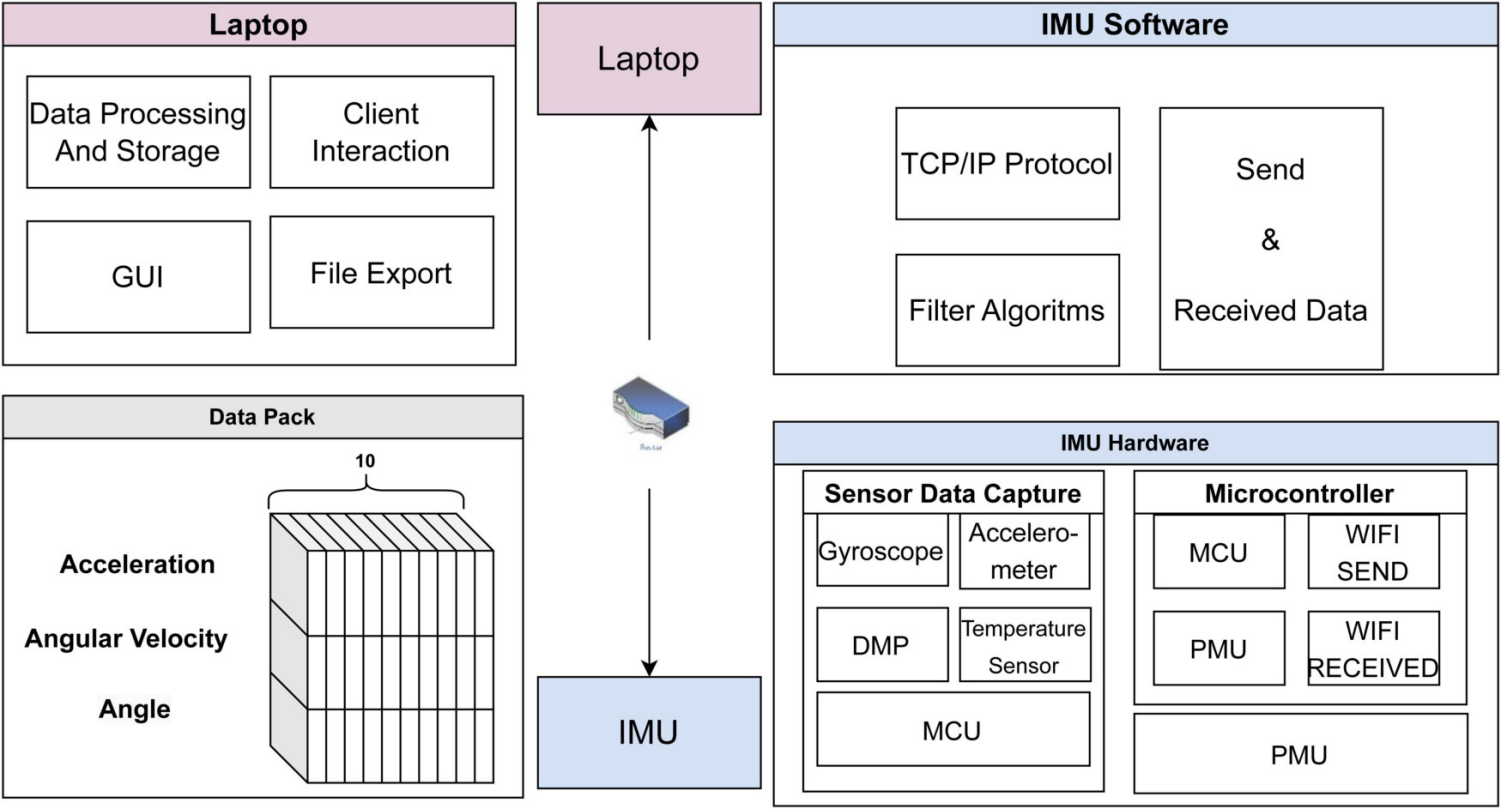
Overall architecture of the IMU-based motion capture system.

The hardware design centers on developing an adaptable, scalable, and portable IoT-based IMU system capable of precise motion capture. Complementing this, the software architecture focuses on efficient real-time data transmission, processing, and classification using advanced deep learning methods. An overview of the IMU system architecture is provided in Figure 1.

Figure 1 presents the overall architecture of the IMU-based motion capture system, comprising hardware, software, and data-processing components. The wearable IMU device integrates a gyroscope, accelerometer, a digital motion processor (DMP), and a temperature sensor to capture multidimensional motion. A microcontroller unit (MCU) handles data acquisition and Wi-Fi transmission, communicating with a server running on a laptop.

On the software side, the IMU-based motion capture system employs TCP/IP protocols and filtering algorithms to manage data transmission and preprocessing. The laptop serves as both the data-processing hub and the user interface, supporting real-time client interaction, data visualization, and export capabilities. Motion data, including acceleration, angular velocity, and angle, is packaged into structured frames, aggregated, and transmitted efficiently to ensure reliable communication and storage for online and offline analysis. This integrated framework enables robust, real-time motion monitoring and provides flexible pathways for subsequent analysis and interpretation. The following subsections describe the hardware configuration, software architecture, and model design for HAR.

### 2.2 Hardware design

**Figure 2:**
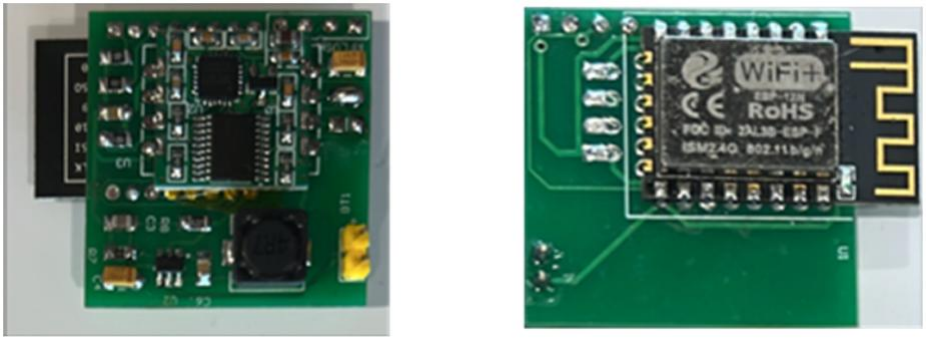
A custom-made circuit board design for an IoT IMU sensor combining an ESP8266 microcontroller, a JY61 sensor used for motion data capture, and experimental work.

The custom-made IoT IMU and data streaming system (Figure 1) consists of two components: the IoT IMU hardware board and the software system, as detailed in this paper. For the realization of the open-source API tailored for human motion detection, a hardware configuration has been designed to meet the following specifications:

1. Physical layout design of the IMU hardware circuitry (Figure 2) is optimized for human motion detection, as well as to provide unobtrusive designs for wearable and equipment-attached sensor housings.
2. Component selection had to support wireless data transmission, high resolution, and transmission sampling rates (up to 100 samples per second) to capture acceleration, angle, and angular velocity data in three dimensions.
3. Reliable wireless transmission of movement data to a designated server.

The design of the IoT IMU sensor incorporates integral hardware components, i.e., ESP8266 as the primary MCU with Wi-Fi module, the JY61 sensor module, which integrates the MPU6050 gyroscope and accelerometer, and a dedicated power supply control module with a detachable and rechargeable 18,650 Li-ion battery. To facilitate the seamless reception of data at the server end, the prototype system in our experimental work was paired with the NETGEAR ROUTER JNDR3000, operating in conjunction with a consumer-grade laptop computer. The dimensions of the IMU wearable device (Figure 2) are 76.34 mm in length, 20.80 mm in width, and 25.40 mm in height, with a weight of approximately 63.7 g, including the battery. The gyroscope functionality of the system offers a full range of $\pm 2000^{\circ }$/s, with a sensitivity of 131 LSB${/}^{\circ }$/s and a noise level of 0.005 $\mathrm{mdps}/\sqrt{\mathrm{Hz}}$. The resolutions for acceleration and angular velocity are $6.1\times 10^{-5}$ g and $7.6\times {10^{-3}}^{\circ }$/s, respectively. Stability metrics indicate that acceleration remains at 0.01 g and angular velocity at $0.05^{\circ }$/s. Additionally, attitude stabilization measurement achieves a precision of $0.01^{\circ }$. The data output frequency is set to 100 Hz with a baud rate of 115,200 bits per second. The accelerometer’s performance spans ±16 g and has a sensitivity of 16,384 LSB/g. As shown in Figure 2, a card-like design is intended to be both portable and wearable on the wrist or other parts of the body or equipment.

### 2.3 Software design

The system code is divided into two main components. The first runs on wearable sensor devices, handles incoming sensor data via the serial port, and controls the microcontroller to transmit it to the server. The server, typically a computer or laptop, receives and parses the incoming data. For each frame, the sensor module sends three sequential data packets to the microcontroller: an acceleration packet, an angular velocity packet, and an angle packet. The transmission operates at a baud rate of 115,200.

The IMU operates at a sampling rate of 100 Hz, which is deliberately selected to balance temporal resolution and computational efficiency. This rate provides sufficient granularity to capture both coarse daily activities (e.g., walking, standing) and fine-grained biomechanical patterns (e.g., golf swing errors), while remaining compatible with the SKS-Transformer’s window-based temporal modeling. Given the model’s input window length of 200 samples, this configuration corresponds to a 2 s temporal receptive field, enabling attention mechanisms to simultaneously capture short-term motion primitives and longer temporal dependencies.

To improve data transmission reliability, we enhanced the microcontroller code to aggregate 10 data packets, each containing acceleration, angular velocity, and angle data, before transmission. This modification effectively reduces the transmission frequency from 100 packets per second to 10 packets per second. The data are transmitted in hexadecimal format, with each value split into sequential low and high bytes that combine to form a signed short integer. For example, as shown in Figure 1 (Data Pack section), Equation 1 shows the *x*-axis acceleration (Ax), which is transmitted as two bytes: AxL (low byte) and AxH (high byte).

Ax=((AxH≪8)∣AxL)÷32,768×16g (1)

To improve temporal consistency and reduce wireless transmission jitter, raw IMU readings are aggregated into packets of 10 consecutive frames before transmission. This packet aggregation strategy lowers the effective transmission frequency while preserving the original sampling resolution. From a modeling perspective, this design reduces irregular inter-arrival times and mitigates network-induced noise, resulting in more temporally stable input sequences that are better suited for attention-based learning.

The proposed HAR system integrates hardware and software components, enhanced with machine learning algorithms, to enable real-time motion data collection and autonomous operation. The hardware design focuses on developing an adaptable, scalable, and portable IoT-based IMU system capable of precise motion capture. On the software side, a Java-based framework manages data reception and control, combining a server backend with a GUI to streamline data acquisition and management. Notably, the software employs multi-threading, enabling concurrent handling of multiple sensor data streams and thereby enhancing the system’s scalability and robustness. The integrated development framework of the HAR system supports real-time data processing and IMU signal visualization, and provides flexible export options, including CSV and log files, to facilitate downstream analysis in programming languages such as Python, Java, and MATLAB.

### 2.4 Selective kernel and squeeze-enhanced axial attention transformer

The **S**elective **K**ernel and **S**queeze-enhanced Axial Attention Transformer (SKS-Transformer) is a deep learning architecture developed for HAR using wearable sensor data. It integrates a sophisticated data-preprocessing pipeline with advanced attention mechanisms to capture both local and global temporal dynamics.

#### 2.4.1 Overall architecture

**Figure 3:**
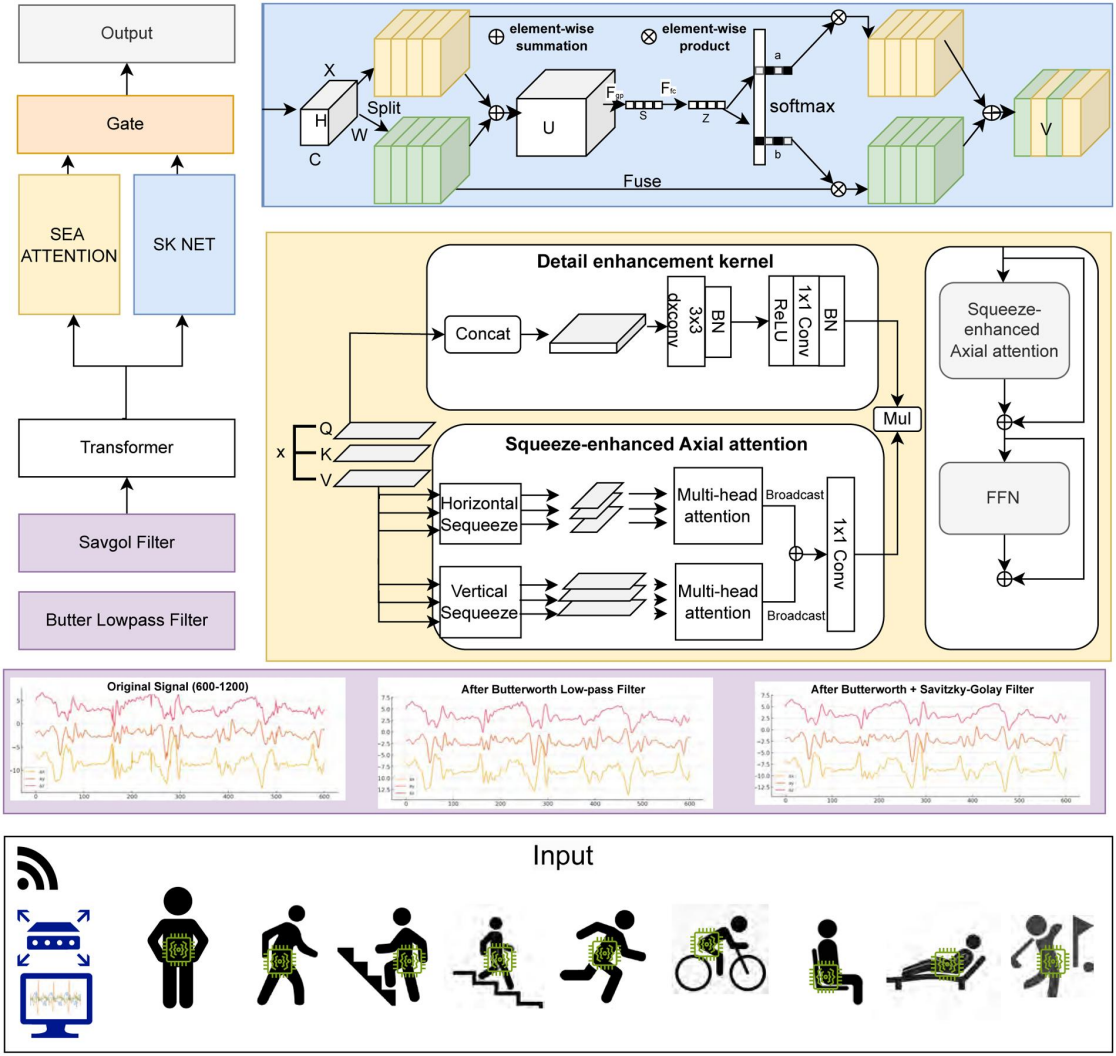
SKS-Transformer architecture.

The overall architecture of the proposed SKS-Transformer is presented in Figure 3. The input comprises denoised, segmented, and normalized accelerometer signals, which are projected into a high-dimensional latent space before being fed into the transformer encoder.

The SKS-Transformer builds on a multi-layer transformer encoder for long-range temporal modeling, further enhanced by two specialized attention modules: the Selective Kernel Networks (SKNet), which extracts multi-scale local features, and the Squeeze-Enhanced Axial (SEA) Attention, which captures directional and contextual dependencies. A gated fusion mechanism dynamically balances these two representations, adaptively weighting local and global information. Finally, a global pooling layer distills the temporal sequence into a compact feature vector, which is passed through a classification layer to produce activity predictions. The entire model is trained end-to-end using cross-entropy loss, enabling joint optimization of all components and achieving robust recognition across diverse human activities. The details of the proposed SKS-Transformer are given in the following subsections.

#### 2.4.2 Input data filtering module

To ensure robust, generalizable model performance, we incorporate an input filtration module that includes signal denoising, window segmentation, label filtering, and normalization. This module is designed to enhance signal quality, enforce label consistency, and stabilize learning, serving as an essential upstream component of the overall model architecture.

#### 2.4.3 Signal denoising

Raw accelerometer signals are inherently noisy due to device jitter, environmental factors, and variations in user-specific movement. To mitigate this, we employed a two-stage denoising process combining frequency-domain filtering with polynomial-based smoothing:

##### 2.4.3.1 Butterworth low-pass filtering

A fourth-order Butterworth filter was applied with a cutoff frequency of 2.5 Hz and a sampling rate of 20 Hz, aiming to remove high-frequency artifacts that are typically unrelated to human motion patterns. This choice is grounded in prior findings that most human activities generate dominant frequency components below 3 Hz. The filter was applied separately to each of the three axes (x,y,z) using a zero-phase forward–backward filter to avoid phase distortion.

##### 2.4.3.2 Savitzky–Golay smoothing

After low-pass filtering, the signal was further refined using a Savitzky-Golay filter with a window length of 11 and a polynomial order of 3. This filter fits a low-degree polynomial to local subsets of the signal using least-squares regression, thereby reducing residual noise while preserving important structural features such as peaks and inflection points.

This denoising strategy improves the signal-to-noise ratio, ensuring the model focuses on meaningful patterns in user activity rather than incidental noise.

#### 2.4.4 Window segmentation

To convert the continuous time-series data into a structured format suitable for supervised learning, we segmented the filtered signals into overlapping sliding windows. Each window contained 200 time steps, equivalent to 10 s of activity, assuming a sampling rate of 20 Hz. A stride of 100 time steps was used to increase the number of training samples and provide temporal continuity across windows.

This window-based approach enables the model to learn short-term temporal patterns while maintaining a fixed input size, which is a requirement for transformer-based architectures.

#### 2.4.5 Label consistency enforcement

To ensure the semantic integrity of each window, we adopted a strict rule: only windows in which all time steps shared the same activity label were retained for training. This constraint eliminates ambiguous samples in which transitions between activities occur within a single window, which could otherwise confuse the classifier and degrade performance.

The activity labels were subsequently encoded as numerical class IDs using a LabelEncoder to prepare them for use in the classification loss function.

#### 2.4.6 Normalization

As a final step, all segmented windows were standardized using z-scores. Specifically, the raw accelerometer readings for each axis (x,y,z) were flattened and standardized to have a zero mean and unit variance. The standardized data were reshaped back to their original window format, with shape (*N*, 200, 3), where *N* is the number of samples.

Normalization ensures that all input features contribute equally to the learning process, which is particularly important in deep learning architectures sensitive to scale differences across input dimensions.

#### 2.4.7 Input projection layer

The input projection layer serves as a bridge between the sensor data and the transformer (Figure 3). This module transforms raw accelerometer signals into a richer, higher-dimensional representation, enhancing their expressiveness and alignment with the model’s internal feature space. Let the input accelerometer signal for a single sample be represented by the matrix

$$X\in R^{T\times D},$$ (2)

where T denotes the number of temporal steps within an input window, and D represents the number of sensor channels. In this work, each window consists of T=200 consecutive sensor readings, and D=3 corresponds to the three accelerometer axes (*x*, *y*, *z*).

To increase the representational capacity of the network and align the input to the transformer dimension, we first project the input into a higher-dimensional latent space $H_{0}$ by using a learnable linear transformation:

$$H_{0}=X\cdot W_{p}+b_{p},$$ (3)

where $W_{p}\in R^{D\times d_{\text{\_}model}}$ and $b_{p}\in R^{d_{\text{\_}model}}$ are trainable parameters. This layer enables the model to transition from sensor features to a semantic latent space, serving as the input embedding for subsequent layers. The input projection process is formally defined in Equations 2 and 3.

#### 2.4.8 Transformer encoder for temporal modeling

To model the temporal dynamics within each input window, we employ a multi-layer transformer encoder (Figure 3). This architecture is particularly effective at capturing long-range temporal dependencies through its global receptive field, which is essential for recognizing complex activity patterns that unfold over multiple seconds. Each encoder layer is composed of two main components: a multi-head self-attention (MHSA) mechanism, which enables the model to attend to relevant information across all time steps, and a position-wise feed-forward network (FFN), which applies non-linear transformations independently at each time step. Both components are integrated with residual connections and layer normalization LayerNorm(⋅) to improve training stability and representation learning. The computations of the transformer encoder, including MHSA and FFN, are described in Equations 3–8.

$$\begin{array}{cc} H^{\prime } & =\text{LayerNorm}(H_{0}+\text{MHSA}(H_{0})),\\ H_{1} & =\text{LayerNorm}(H^{\prime }+\text{FFN}(H^{\prime })). \end{array}$$ (4)

Specifically, the MHSA module first computes query, key, and value matrices through learned linear projections:

$$Q=H_{0}W^{Q},\;K=H_{0}W^{K},\;V=H_{0}W^{V},$$ (5)

where $W^{Q},W^{K},W^{V}\in R^{d_{\text{\_}model}\times d_{k}}$ are trainable projection matrices, and $d_{k}$ is the attention head dimension as denoted in Figure 3. Scaled dot-product attention is computed as:

$$\text{Attention}(Q,K,V)=\text{softmax}\left(\frac{QK^{⊤}}{\sqrt{d_{k}}}\right)V.$$ (6)

Multiple heads (say h heads) are computed in parallel, concatenated, and projected:

$$\text{MHSA}(H_{0})=\text{Concat}({\text{head}}_{1},\ldots ,{\text{head}}_{h})W^{O},$$ (7)

where $W^{O}\in R^{hd_{k}\times d_{\text{\_}model}}$ is the output projection matrix. This allows the model to capture diverse temporal patterns and long-range temporal dependencies. The FFN (as denoted in Figure 3) is applied independently at each time step and consists of two fully connected layers with a non-linear activation:

$$\text{FFN}(h_{t})=\text{ReLU}(h_{t}W_{1}+b_{1})W_{2}+b_{2},$$ (8)

where $h_{t}\in R^{d_{\text{\_}model}}$ is the input at timestep t, $W_{1}\in R^{d_{\text{\_}model}\times d_{\text{\textbackslash{},ff}}}$, $W_{2}\in R^{d_{\text{\textbackslash{},ff}}\times d_{\text{\_}model}}$, and $d_{\text{\textbackslash{},ff}}$ is the FFN hidden dimension. This component enhances the model’s capacity by applying non-linear transformations and cross-dimensional interactions at each time step.

We stack two such encoder layers in our architecture, enabling the model to progressively refine temporal dependencies through hierarchical attention.

#### 2.4.9 Selective Kernel networks

Selective Kernel Networks (SKNet) is a dynamic feature selection mechanism that enables the model to adaptively choose between multiple receptive field sizes, allowing it to focus on the most relevant temporal scales for a given input (32). To enrich the temporal representation with multi-scale local features, we introduce an SKNet module. The operations of the Selective Kernel module are mathematically formulated in Equations 9–14. The module applies multiple grouped convolutional operations in parallel with varying kernel sizes k∈{1,3,5,7}, each capturing patterns at different temporal resolutions:

$$U_{k}=\text{ReLU}\left(\text{BN}\left({\text{Conv}}_{k}(H_{1})\right)\right),\;\forall k.$$ (9)

Here, $U_{k}\in R^{T\times d_{\text{\_}model}}$ is the output of the k-th convolution branch after batch normalization (BN) and ReLU(⋅) activation function (as denoted in Figure 3). We then aggregate all branch outputs by summation:

$$U=\sum_{k}U_{k}.$$ (10)

Next, we compute a global context descriptor z via temporal average pooling over all timesteps:

$$z=\frac{1}{T}\sum_{t=1}^{T}U_{t}.$$ (11)

This descriptor is passed through a shared fully connected layer with ReLU(⋅) activation function to generate a compact selection vector:

$$s=\text{ReLU}(W_{1}z).$$ (12)

Here, $W_{1}\in R^{d_{\text{\_}model}\times d_{s}}$ is a trainable weight matrix that maps the global context vector z to a lower-dimensional selection vector s, where $d_{s}$ is the hidden dimension of the selection module. For each branch, we calculate an attention weight:

$$\alpha_{k}=\frac{\exp(w_{k}^{⊤}s)}{\sum_{j}\exp(w_{j}^{⊤}s)},$$ (13)

where $w_{k}$ is the parameter vector associated with the k-th branch, and $\alpha_{k}$ denotes its normalized importance score. Finally, we fuse the multi-scale features by weighting each branch output with its corresponding attention score:

$$V_{\text{SK}}=\sum_{k}\alpha_{k}\cdot U_{k}.$$ (14)

This selective fusion enables the model to dynamically adapt to the temporal scales most relevant to each input, thereby improving its generalization across diverse activity patterns.

#### 2.4.10 Squeeze-enhanced axial attention

To efficiently capture directional dependencies and global contextual information, we incorporate a Squeeze-enhanced Axial Attention module (Figure 3) inspired by SeaFormer (33). This module decomposes self-attention into two orthogonal components:

1. Row-wise Axial Attention, capturing temporal dependencies across time steps, and
2. Column-wise Axial Attention, modeling inter-channel relationships.

The squeeze-enhanced axial attention mechanism is defined in Equations 15–19. Given a 4D input tensor $X\in R^{B\times C\times H\times W}$ (produced by the transformer encoder), we first generate query, key, and value tensors via depthwise convolution. To reduce computation, we apply mean pooling along one axis to squeeze the spatial dimension:

$$Q_{\text{row}}={\text{PosEmb}}_{\text{row}}\left(\frac{1}{W}\sum_{w=1}^{W}Q_{\;:\;,\;:\;,\;:\;,w}\right),\;K_{\text{row}}={\text{PosEmb}}_{\text{row}}\left(\frac{1}{W}\sum_{w=1}^{W}K_{\;:\;,\;:\;,\;:\;,w}\right).$$ (15)

Similarly, column attention is computed using mean pooling over H:

$$Q_{\text{col}}={\text{PosEmb}}_{\text{col}}\left(\frac{1}{H}\sum_{h=1}^{H}Q_{\;:\;,\;:\;,h,\;:\;}\right),\;K_{\text{col}}={\text{PosEmb}}_{\text{col}}\left(\frac{1}{H}\sum_{h=1}^{H}K_{\;:\;,\;:\;,h,\;:\;}\right).$$ (16)

Attention maps are calculated as:

$$A_{\text{row}}=\text{Softmax}\left(\frac{Q_{\text{row}}\cdot K_{\text{row}}^{⊤}}{\sqrt{d_{k}}}\right),\;A_{\text{col}}=\text{Softmax}\left(\frac{Q_{\text{col}}\cdot K_{\text{col}}^{⊤}}{\sqrt{d_{k}}}\right).$$ (17)

The outputs are then processed with depthwise convolutional encoders:

$$O_{\text{row}}={\text{Conv}}_{\text{row}}(A_{\text{row}}\cdot V_{\text{row}}),\;O_{\text{col}}={\text{Conv}}_{\text{col}}(A_{\text{col}}\cdot V_{\text{col}}).$$ (18)

Finally, the attended values are fused with the original input via a residual connection and projected with non-linearity:

$$V_{\text{SEA}}=\sigma \left(\text{Proj}\left(V+O_{\text{row}}+O_{\text{col}}\right)\right).$$ (19)

This design effectively encodes structured long-range dependencies in both temporal and channel dimensions, while maintaining high efficiency via axial factorization and squeeze operations.

#### 2.4.11 Gated fusion mechanism

To dynamically balance local and global feature representations, we introduce a lightweight Gated Fusion that adaptively integrates outputs from the Selective Kernel Attention and the Squeeze-Enhanced Axial Attention modules. This design enables the model to selectively emphasize either multi-scale local patterns or directional global dependencies, depending on the input sequence.

Given the feature maps produced by the SKNet module ($V_{\text{SK}}\in R^{B\times C\times T\times 1}$) and the SeaAttention module ($V_{\text{SEA}}\in R^{B\times C\times T\times 1}$), we generate a dynamic gating signal $G\in [0,1{]}^{B\times 1\times T\times 1}$ via a parameter-efficient 1×1 convolution followed by a sigmoid activation. The gating signal is computed from the original transformer-encoded features before attention, guiding the fusion process based on raw global temporal context. The gated fusion process is formally described in Equations 20 and 21. Formally:

$$G=\sigma \left({\text{Conv}}_{1\times 1}\left(X_{\text{transformed}}\right)\right),$$ (20)

where σ(⋅) denotes the sigmoid activation and $X_{\text{transformed}}\in R^{B\times C\times T\times 1}$ represents the intermediate feature map output from the transformer encoder.

The two attention pathways are then adaptively fused via a convex combination controlled by the learned gating mask:

$$V_{\text{\textbackslash{},fused}}=G\cdot V_{\text{SK}}+(1-G)\cdot V_{\text{SEA}},$$ (21)

where the gating mask G functions as a soft selector, dynamically controlling the contribution of each attention branch at every temporal step and across all feature channels. When G→1, the model prioritizes local multi-scale features captured by the SKNet module; conversely, when G→0, the global directional dependencies modeled by the SeaAttention module are emphasized.

This adaptive fusion strategy enables the network to dynamically balance local and global features based on the input, enhancing feature diversity and preventing overreliance on any single attention branch. By selectively integrating complementary information, it produces efficient, expressive representations without incurring significant computational overhead. Notably, the gating operation introduces minimal computational overhead due to its compact 1×1 convolution design while significantly enhancing the model’s expressive power.

#### 2.4.12 Global pooling and classification

After the gated fusion of multi-scale local and global contextual features, the resulting feature map $V_{\text{\textbackslash{},fused}}\in R^{B\times C\times T\times 1}$ is processed via global average pooling to aggregate temporal information into a compact global descriptor. Specifically, temporal global average pooling is applied along the sequence length dimension:

$$h_{\text{\textbackslash{},pool}}=\frac{1}{T}\sum_{t=1}^{T}V_{\text{\textbackslash{},fused}}[\;:\;,\;:\;,t,\;:\;],$$ (22)

yielding a global representation $h_{\text{\textbackslash{},pool}}\in R^{B\times C}$ for each input sample. Subsequently, this global feature vector is projected into the activity label space using a fully connected classification layer:

$$\hat{y}=W_{o}\cdot h_{\text{\textbackslash{},pool}}+b_{o},$$ (23)

where $W_{o}\in R^{C\times K}$ and $b_{o}\in R^{K}$ are the learnable weights and bias of the classifier, and K denotes the number of activity classes.

Finally, the class probabilities p are obtained via a softmax activation function:

$$p=\text{Softmax}(\hat{y}).$$ (24)

This classification head enables end-to-end training of the entire model with a standard cross-entropy loss, facilitating joint optimization of feature extraction and decision-making. The global pooling and classification operations are defined in Equations 22–24.

## 3 Results

In this section, we present three experiments designed to evaluate both the proposed SKS-Transformer model and the complete IMU-based motion capture pipeline for our real-world data. Specifically, Experiment 1 assesses the proposed model’s performance on standard HAR benchmark datasets, while Experiment 2 evaluates the end-to-end system using data collected through our IMU-based motion capture setup. Experiment 3 provides an ablation study to analyze the contributions of key model components.

### 3.1 Datasets and experimental setup

We evaluate the proposed model on four datasets: two standard HAR benchmarks (UCI HAR and PAMAP2) and two custom datasets collected with our IMU-based motion capture system, i.e., an in-house HAR dataset and a golf swing error dataset. The benchmark datasets evaluate the model’s general performance, while the custom datasets assess its real-world applicability and domain-specific capabilities. For a fair comparison, we evaluate our approach against established baseline architectures using consistent training and evaluation protocols across all datasets.

**Figure 4:**
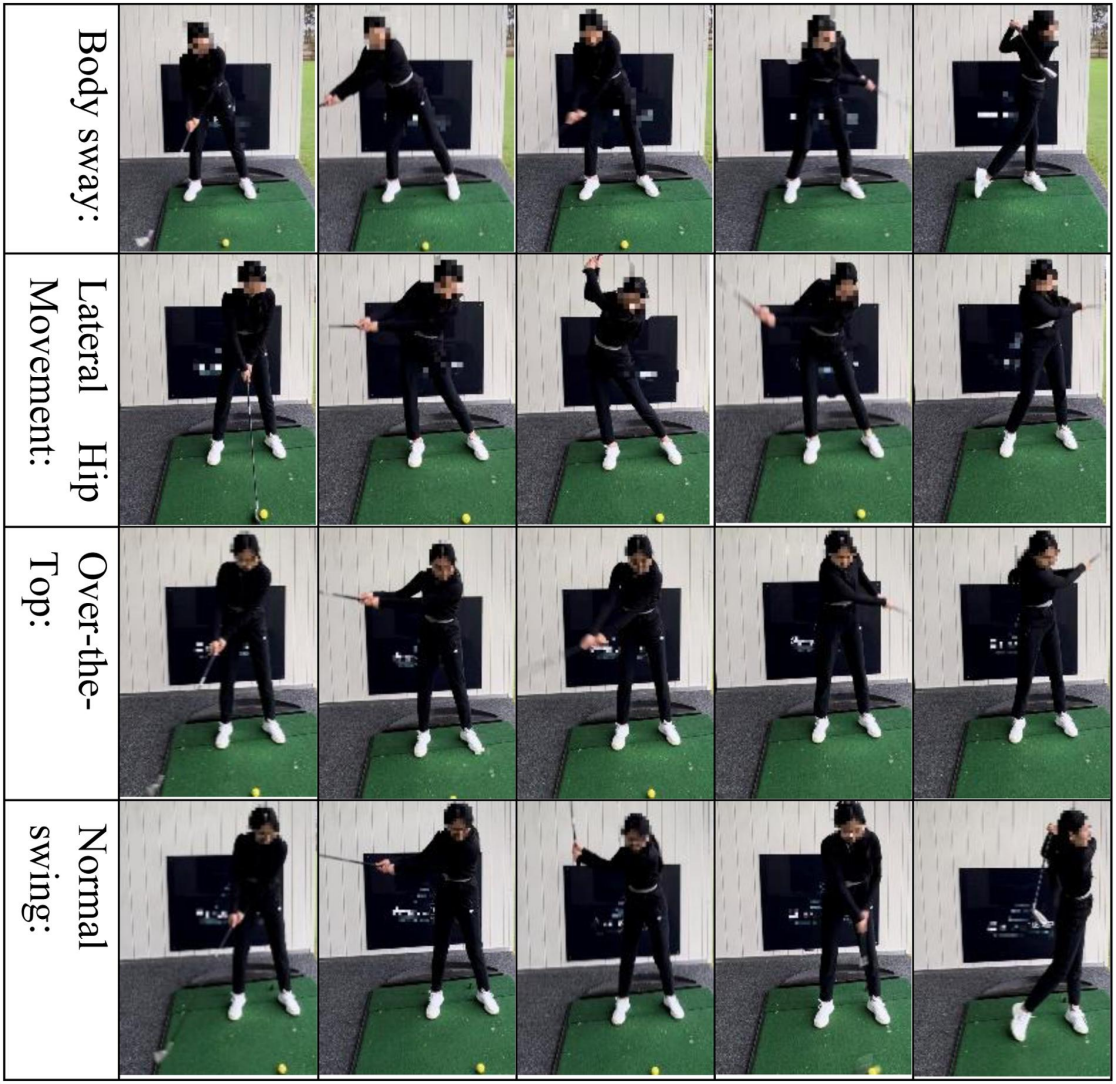
Common golf swing mistakes and normal swings recorded with a degree of point-of-view (POV) distortion.

- **The UCI dataset:** (34)^1^ This benchmark dataset contains sensor recordings from 30 participants (aged 19–48) performing six daily activities: walking, walking upstairs, walking downstairs, sitting, standing, and lying down. Each participant wore a waist-mounted Samsung Galaxy S II smartphone that captured 3-axis accelerometer and gyroscope data at 50 Hz. Video recordings were used to ensure accurate labeling of activities.
- **The PAMAP2 dataset:** (35)^2^ The PAMAP2 dataset comprises recordings from 9 subjects participating in 18 diverse physical activities, including walking, cycling, rope jumping, and soccer. Each subject was equipped with three wireless IMUs placed on the dominant wrist, chest, and dominant ankle, as well as a heart rate monitor. IMU data were sampled at 100 Hz and included accelerometer, gyroscope, and magnetometer signals, while heart rate data were recorded at approximately 9 Hz. Data collection followed a standardized activity protocol.
- **In-house HAR dataset:** This custom dataset comprises six daily activities commonly observed in real-world settings: walking, running, walking downstairs, walking upstairs, standing, and marching in place. The data were collected using a custom-made IMU-based wearable sensing system (developed by the first author, as described in Section 2.2) that captures detailed motion signals for human activity recognition. The IMU-based wearable sensor was worn on the waist, a commonly used location for capturing whole-body motion dynamics. The recorded signals included triaxial linear acceleration, angular velocity, and orientation angles from the JY61 sensor.
- **Golf swing error dataset:** This domain-specific dataset is designed to evaluate fine-grained motion classification in athletic training contexts, focusing on golf swing performance. The data were also collected using a custom-made IMU-based wearable sensing system (described in Section 2.2), with the sensor mounted on the waist to capture detailed kinematic features during each swing. Figure 4 provides visual illustrations of these swing categories, emphasizing how common golf swing mistakes and normal swings may be perceived differently under point-of-view (POV) distortions. Each category contains 30 labeled samples, ensuring class balance for model training and evaluation. The collected dataset includes four labeled swing categories as follows:
  - **Normal swing.** A technically correct swing commonly seen in intermediate to advanced players, characterized by proper alignment, balance, and coordinated mechanics, resulting in a straight and consistent ball flight.
  - **Lateral hip movement.** Swings that exhibit excessive side-to-side motion of the hips during the downswing or follow-through often lead to a loss of balance and reduced strike consistency.
  - **Body sway.** Swings where the upper body shifts excessively backward during the backswing or forward during the downswing, disrupting posture and negatively impacting shot stability and directional control.
  - **Over-the-top.** A common swing fault where the club moves outside the ideal swing plane during the transition from backswing to downswing, typically resulting in a steep attack angle and a left-to-right (slice) ball flight.

The erroneous golf swing categories were defined based on expert judgment and established coaching principles. Specifically, “Lateral Hip Movement” refers to visually observable excessive lateral displacement of the hips during backswing and downswing; “Body Sway” denotes pronounced backward or forward upper-body shift relative to the stance; and “Over-the-Top” describes an outward club path during the transition from backswing to downswing. These labels were assigned based on repeated visual inspection and consistency across trials, rather than strict quantitative thresholds.

#### 3.1.1 Experimental setup

All models are trained and evaluated under identical experimental conditions, using the same inertial measurement unit (IMU) dataset collected with our previously described custom-built IoT IMU sensor system (combining ESP8266 microcontroller and JY61 sensor). The HAR and Golf Swing datasets provide a consistent input format across all models, allowing for a fair and controlled comparison. The key hardware equipment and experimental configuration for modeling and analysis are listed in Table 1.

**Table 1:** Hardware configuration and settings for experimental modeling and analysis.

Setting	Value
Framework	PyTorch
Hardware	NVIDIA Tesla V100 GPU
Optimizer	Adam
Learning rate	0.001
Loss function	CrossEntropyLoss
Batch size	64
Normalization	Z-score standardization

#### 3.1.2 Baseline models and comparisons

To ensure a fair and comprehensive evaluation, we benchmark the proposed SKS-Transformer against representative HAR architectures spanning the commonly-known original models (e.g., CNN, GRU, RCNN, LSTM, BiLSTM, and Transformer) as well as more complex CNN–based hybrids and attention/ensemble designs. The more complex baseline models used in the experimental work are summarized as follows:

- **Multi-input CNN–GRU** (23): Parallel CNN streams process modality- or axis-specific inputs (e.g., accelerometer and gyroscope), whose features are concatenated and fed to a GRU for temporal aggregation. This design prioritizes modality-specific encoding prior to sequence modeling.
- **CNN–LSTM** (13): A canonical hybrid pipeline where 1D CNN layers extract local temporal patterns and an LSTM layer models long-range dependencies. This serves as a strong, computation-conscious mixed baseline.
- **Multi-branch CNN–BiLSTM** (36): Multiple CNN branches with different kernel sizes perform multi-scale feature extraction; a BiLSTM layer then integrates bidirectional temporal context, improving robustness to pace variations and duration differences.
- **CNN–BiLSTM + Attention** (37): A parallel CNN/BiLSTM backbone followed by a soft temporal attention module that reweights critical timesteps, particularly helpful under noisy/missing samples.
- **Ensem-HAR** (38): A stacking ensemble that trains several backbones (e.g., CNN, CNN–LSTM, ConvLSTM, Stacked-LSTM) and learns a meta-learner (“blender”) to fuse their outputs, trading extra computation for improved stability across datasets.

### 3.2 Experiment 1: model evaluation on standard HAR benchmark datasets

In the first experiment, we evaluate SKS-Transformer on two public HAR benchmarks, i.e., UCI HAR and PAMAP2, using 5-fold cross-validation and standardized training/evaluation protocols. We first report per-fold results and then compare them against strong baselines trained under identical settings.

#### 3.2.1 UCI HAR (5-fold cross-validation)

As shown in Table 2, SKS-Transformer attains an average accuracy of 96.80% (F1: 97.01%, precision: 97.03%, recall: 97.02%), with fold accuracies ranging from 96.26% to 97.23%. Precision and recall are closely aligned, indicating balanced errors and stable generalization across splits.

**Table 2:** 5-Fold cross-validation performance on UCI HAR dataset.

Fold	Accuracy (%)	F1 score (%)	Precision (%)	Recall (%)
1	96.99	97.19	97.19	97.19
2	96.50	96.71	96.70	96.76
3	97.23	97.42	97.42	97.41
4	97.04	97.24	97.29	97.28
5	96.26	96.49	96.55	96.47
**Average**	**96.80**	**97.01**	**97.03**	**97.02**

Bold values represent the average results obtained from five-fold cross-validation.

#### 3.2.2 UCI HAR: comparison with baselines

Table 3 summarizes results against recurrent, convolutional, and hybrid/attention models. While several commonly-known original models (e.g., CNN, LSTM, GRU, and Transformer) perform in the approximate 70%–92% range, hybrid and attention-based baselines improve substantially [best baseline: 96.71% accuracy from CNN-based Bi-LSTM + Attention (37)]. Nonetheless, SKS-Transformer demonstrates leading accuracy and consistently strong performance across evaluation metrics.

**Table 3:** Comparison of HAR models on UCI-HAR dataset.

Model	Accuracy	F1-measure	Precision	Recall
CNN	0.7065	0.7179	0.6982	0.6938
GRU	0.8778	0.8791	0.8752	0.8761
RCNN	0.9196	0.9175	0.9170	0.9170
LSTM	0.8521	0.8519	0.8474	0.8481
BiLSTM	0.7981	0.8114	0.7931	0.7942
Transformer	0.9203	0.9194	0.9266	0.9177
Multi-input CNN-GRU (23)	0.9620	0.9602	0.9589	0.9617
CNN-LSTM (13)	0.9213	0.9188	0.9179	0.9198
Multi-branch CNN-BiLSTM (36)	0.9637	0.9613	0.9599	0.9628
CNN-based Bi-LSTM + Attention (37)	0.9671	0.9614	0.9619	0.9611
Ensem-HAR (38)	0.9505	0.9552	0.9509	0.9597
**Proposed SKS-Transformer Model**	**0.9701**	**0.9680**	**0.9703**	**0.9702**

Bold values represent the average results obtained from five-fold cross-validation.

#### 3.2.3 PAMAP2 (5-fold cross-validation)

PAMAP2 covers 18 activities with high-frequency, multi-IMU signals. In Table 4, SKS-Transformer reaches an average accuracy of 98.00% (F1: 97.85%, precision: 98.13%, recall: 97.84%), with fold accuracies between 95.89% and 99.54%. The tight alignment between precision and recall again suggests class-balanced performance.

**Table 4:** Cross-validation performance metrics on PAMAP2 dataset.

Fold	Accuracy	F1 score	Precision	Recall
1	97.73	97.59	98.04	97.25
2	97.27	97.09	97.16	97.14
3	99.54	99.39	99.51	99.31
4	99.54	99.64	99.68	99.62
5	95.89	95.59	96.23	95.87
**Average**	**98.00**	**97.85**	**98.13**	**97.84**

Bold values indicate the performance of our proposed model.

#### 3.2.4 PAMAP2: comparison with baselines

As reported in Table 5, conventional CNN/LSTM/GRU architectures achieve 88.69%–94.05% accuracy while stronger hybrid/attention models reach up to 97.91% [Ensem-HAR from (38)]. Again, SKS-Transformer outperforms all compared approaches with 98.00% accuracy (F1: 97.85%, precision: 98.13%, recall: 97.84%). Although the margin over the strongest baseline is modest on this saturated benchmark, the gains are consistent across metrics, supporting the benefits of multi-scale feature extraction and direction-aware attention.

**Table 5:** Performance comparison of various models on PAMAP2 dataset.

Model	Accuracy	F1-score	Precision	Recall
CNN	0.9345	0.9299	0.9477	0.9182
GRU	0.8869	0.8623	0.8951	0.8419
RCNN	0.9286	0.9348	0.9572	0.9170
LSTM	0.8929	0.8920	0.8851	0.9071
BiLSTM	0.9107	0.8695	0.9241	0.8541
Transformer	0.9405	0.9328	0.9254	0.9416
Multi-input CNN-GRU (23)	0.9494	0.9494	0.9523	0.9494
CNN-LSTM (13)	0.9405	0.9388	0.9416	0.9405
Multi-branch CNN-BiLSTM (36)	0.9524	0.9523	0.9541	0.9524
CNN-based Bi-LSTM + Attention (37)	0.9583	0.9574	0.9581	0.9583
Ensem-HAR (38)	0.9791	0.9793	0.9791	0.9791
**Proposed SKS-Transformer Model**	**0.9800**	**0.9785**	**0.9813**	**0.9784**

Bold values indicate the performance of our proposed model.

Table 5 summarizes the performance of the proposed SKS-Transformer compared with a range of baseline and state-of-the-art models on the PAMAP2 dataset. Conventional architectures such as CNN, LSTM, and GRU achieve relatively moderate performance, with accuracies ranging from 88.69% to 93.45%. Hybrid models that combine convolutional and recurrent layers (e.g., CNN-LSTM, Multi-branch CNN-BiLSTM) and those incorporating attention mechanisms generally achieve higher performance, with the best-performing baseline, Ensem-HAR, reaching 97.91% accuracy. The SKS-Transformer surpasses all compared approaches, achieving the highest accuracy (98.00%), F1-score (97.85%), precision (98.13%), and recall (97.84%). These improvements, though marginal relative to the strongest baseline, indicate that the proposed multi-scale and direction-aware attention design consistently enhances recognition performance, even on challenging multi-activity datasets such as PAMAP2.

### 3.3 Experiment 2: end-to-end system evaluation on real-world sensor-collected data

This experiment evaluates the full pipeline, from our IMU-based wearable data-acquisition system (hardware/firmware) through to SKS-Transformer inference, using two in-house datasets captured entirely with our motion-capture setup. Different from Experiment 1, which isolates model performance on public benchmarks, we assess system-level performance under real-world operating conditions, jointly reflecting acquisition fidelity and downstream recognition.

#### 3.3.1 Self-collected HAR dataset (end-to-end performance evaluation)

We first assess the pipeline on a general HAR task using data captured by our custom IMU system, training and evaluating all models under identical protocols. As shown in Table 6, conventional models exhibit moderate accuracy (typically <0.90) in unconstrained settings. Hybrid CNN and attention-based baselines improve substantially (best baseline: Ensem-HAR at 0.9524). SKS-Transformer achieves the top results across all metrics (accuracy: 0.9810, F1: 0.9845, precision: 0.9827, recall: 0.9833), indicating effective multi-scale temporal modeling and direction-aware attention for robust real-world recognition.

**Table 6:** End-to-end performance on the self-collected HAR dataset.

Model	Accuracy	F1 score	Precision	Recall
CNN	0.6250	0.5833	0.7656	0.6250
LSTM	0.8600	0.8700	0.9200	0.8600
GRU	0.7100	0.7100	0.8100	0.7100
RCNN	0.8600	0.8700	0.9200	0.8600
BiLSTM	0.7619	0.7730	0.8095	0.7619
Transformer	0.8100	0.8000	0.8200	0.8100
Multi-input CNN-GRU (23)	0.6250	0.5833	0.7656	0.6250
CNN-LSTM (13)	0.9048	0.9048	0.9048	0.9048
Multi-branch CNN-BiLSTM (36)	0.8571	0.8597	0.8690	0.8571
CNN-based Bi-LSTM + Attention (37)	0.9048	0.9028	0.9259	0.9048
Ensem-HAR (38)	0.9524	0.9585	0.9762	0.9524
**Proposed SKS-Transformer Model**	**0.9810**	**0.9845**	**0.9827**	**0.9833**

Bold values indicate the performance of our proposed model.

#### 3.3.2 Golf swing error detection (fine-grained, end-to-end performance evaluation)

We evaluate the end-to-end pipeline on a fine-grained sports task, golf swing error detection, using data captured entirely with our IMU-based acquisition system. As shown in Table 7, several strong baselines exceed 0.98 accuracy (e.g., CNN–LSTM, attention-augmented CNN–BiLSTM, Ensem-HAR), indicating a cleanly separable and well-labeled dataset. SKS-Transformer achieves perfect scores across accuracy, F1 score, precision, and recall, demonstrating sensitivity to subtle biomechanical differences. Taken together, these results suggest that (i) the signals acquired with our sensor platform and labeling protocol are of sufficient quality to support high-accuracy recognition across diverse architectures, and (ii) the end-to-end pipeline appears suitable for sports performance analysis; nevertheless, since these results chiefly reflect the SKS-Transformer’s behavior on signals acquired by our device, they should be interpreted as the joint effect of acquisition and modeling rather than as a hardware-only claim.

**Table 7:** Performance comparison of models on the golf swing error detection dataset.

Model	Accuracy	F1 score	Precision	Recall
CNN	0.9792	0.9791	0.9808	0.9792
LSTM	0.9583	0.9573	0.9615	0.9583
GRU	0.9537	0.9491	0.9558	0.9463
RCNN	0.9907	0.9911	0.9909	0.9915
BiLSTM	0.9792	0.9791	0.9808	0.9792
Transformer	0.9792	0.9791	0.9808	0.9792
Multi-input CNN-GRU (23)	0.9815	0.9814	0.9816	0.9815
CNN-LSTM (13)	0.9907	0.9907	0.9908	0.9907
Multi-branch CNN-BiLSTM (36)	0.9769	0.9767	0.9778	0.9769
CNN-based Bi-LSTM + Attention (37)	0.9861	0.9862	0.9863	0.9861
Ensem-HAR (38)	0.9954	0.9954	0.9954	0.9954
**Proposed SKS-Transformer Model**	**1.0000**	**1.0000**	**1.0000**	**1.0000**

Bold values indicate the performance of our proposed model.

### 3.4 Experiment 3: ablation study

In this experiment, we quantify the contribution of key components by removing them sequentially from the proposed SKS-Transformer model and disabling the input preprocessing filters. Specifically:

- *No_SK* removes the selective-kernel branch (multi-scale pathway).
- *No_SEA* removes the squeeze-enhanced axial attention.
- *No_Gate* disables the gating/fusion between pathways.
- *No_Filter* skips low-pass/smoothing in preprocessing.
- *No_Attn* (golf only) removes attention mechanisms entirely.

#### 3.4.1 Ablation study on PAMAP2 benchmark dataset

To keep the ablation both informative and tractable, we evaluate on two representative datasets: the public *PAMAP2* benchmark (Table 8) and the self-collected golf swing error dataset (Table 9) acquired entirely with our IMU-based hardware. Together, these datasets encompass both generic HAR and fine-grained sports analysis, and we consider this combination sufficient to characterize component contributions and overall performance within the scope of this study.

**Table 8:** Ablation results on *PAMAP2* dataset.

Variant	Accuracy	Precision	Recall	F1 score
FullModel	**0.9800**	**0.9713**	**0.9784**	**0.9785**
No_SK	0.9464	0.9306	0.9371	0.9326
No_SEA	0.9583	0.9533	0.9340	0.9422
No_Gate	0.9226	0.9199	0.9105	0.9116
No_Filter	0.9643	0.9629	0.9519	0.9558

Bold values indicate the performance of our proposed model.

**Table 9:** Ablation results on the golf swing error detection task.

Variant	Accuracy	Precision	Recall	F1 score
FullModel	**1.0000**	**1.0000**	**1.0000**	**1.0000**
No_SK	0.9792	0.9808	0.9792	0.9791
No_SEA	0.9792	0.9808	0.9792	0.9791
No_Gate	0.9792	0.9808	0.9792	0.9791
No_Attn	0.9583	0.9615	0.9583	0.9573

Bold values indicate the performance of our proposed model.

#### 3.4.2 Ablation study on golf swing error detection

Being evaluated on the PAMAP2 dataset, the full model attains the best performance (Acc. 0.9800, F1 0.9785). Removing the gating mechanism yields the largest drop (F1 −0.0669), indicating that *learned fusion* between pathways is critical. Eliminating the SK branch (F1 −0.0459) or SEA attention (F1 −0.0363) also degrades results substantially, underscoring the importance of multi-scale features and direction-aware attention. Skipping input filtering (F1 −0.0227) still hurts performance, showing that noise suppression before modeling remains beneficial.

Being evaluated on the golf swing error detection dataset, the full model achieves perfect scores across all metrics, reflecting strong class separability in this fine-grained task. Removing any single component (SK, SEA, or gating) produces a similar, modest decline (∼2.1 pp in accuracy; F1 −0.0209), suggesting partial redundancy among these mechanisms under this dataset. However, removing *all* attention (*No_Attn*) causes a larger drop (accuracy −4.17 pp; F1 −0.0427), indicating that attention-based feature extraction is essential for reliably capturing the subtle biomechanical differences between correct and erroneous swings.

Across both datasets, performance gains arise from the combination of (i) multi-scale feature extraction (SK), (ii) direction-aware attention (SEA), (iii) gated fusion, and (iv) denoised inputs. Gating accounts for most of PAMAP2; attention is indispensable for the fine-grained golf task overall.

## 4 Discussion

The presented system was designed to be modular, adaptable, low-power, and scalable, supporting accurate motion tracking in both generic daily activities, such as walking, sitting, or stair climbing, and fine-grained motion patterns that involve subtle biomechanical variations, such as distinguishing between correct and faulty golf swings. These HAR scenarios require not only broad temporal modeling but also high sensitivity to localized motion cues. As part of the system, the developed SKS-Transformer model introduces two key innovative features to enhance temporal feature learning in sensor-based HAR: (1) Selective Kernel Networks module designed to dynamically capture the local temporal features at multiple scales. (2) A Squeeze-Axial Attention module, designed to process directional dependencies across both temporal and feature dimensions. Both modules are integrated through a learnable gated fusion mechanism, enabling the model to adaptively balance global and local representations. The SKS-Transformer architecture provides strong expressiveness in modeling complex motion dynamics while maintaining efficiency suitable for deployment in the presented HAR contexts.

For modeling and analysis of various human activities, we evaluated our system on two well-known public sensor-based motion datasets, UCI-HAR^3^ and PAMAP2,^4^ and two additional custom datasets encompassing daily activities and fine-grained golf swing errors collected (by the first author) through our sensor platform. Experimental results demonstrate that on both public datasets, the SKS-Transformer model consistently surpassed the state of the art (by 0.3% and 0.09%) compared to the best of 11 other published models, and on collected HAR datasets by 2.86%, achieving the accuracy of up to 98.10%, and on the golf dataset by 0.46%, achieving 100% accuracy of swing error detection.

Additionally, the developed software pipeline supports real-time data acquisition, preprocessing, storage, and visualization. Coupled with the hardware design, which is based on the ESP8266 microcontroller and JY61 sensor, the system is modular (i.e., allowing integration or substitution with alternative components) and intended to be published as open source, along with supporting documentation for wireless streaming of accelerometer and gyroscope data. The software includes signal denoising, sliding window segmentation, label filtering, and normalization to prepare the input for the SKS-Transformer model. Such a non-proprietary and transparent system design is aimed at democratizing HAR technology for the benefits of society and to incentivize the global research community to advance privacy-preserving systems, which can be deployed locally to meet the end-user requirements.

### 4.1 Limitations

For IoT IMU sensor design, the first obvious choice for prototyping open-source hardware (and software) for HAR would be to start with a modular design relying on a readily available Arduino modular design (and library for JY61/MPU6050 sensor). For custom-made printed circuit board (PCB) design, the chosen low-cost ESP8266 microcontroller has no Bluetooth communication option, but it operates on lower clock speed than e.g., the newer alternative such as ESP32 (with Wi-Fi and Bluetooth), hence making it a compact and desirable design alternative for HAR applications requiring a mix of computational performance, battery size and data capture expectations for real-life situations including daily activities of longer durations. As already criticized, compared to readily available mobile app prototyping (such as Matlab), in our experiments, direct IMU data streaming from the custom-made ESP8266/JY61 sensor does not rely on overseas cloud infrastructures, hence enabling real-life privacy-preserving systems research and development (R&D) and localized/on-premise infrastructure deployments. Other impediments of mobile IMU sensor data acquisitions include:

- Mass of a mobile device (approximate range between 130 g and 250 g) being substantially greater than 70 g IoT IMU sensor (Figure 2), where a lighter IMU sensor introduces less experimental bias and level of obtrusiveness, especially for fine-grained subtle activities monitoring;
- The default mobile device setting is to auto-lock/sleep, while in contrast, our current IMU sensor software (Figure 1) is not configured to switch to power saving sleep mode; and
- For the real-life HAR contexts where a low-cost sensor may be a better alternative than a harder-to-replace mobile option.

Across both public datasets, SKS-Transformer achieved relatively minor improvements in classification accuracy (0.3% and 0.09%) compared with the best of 11 other published models. However, 97.1% and 98.00% could arguably be considered as already high classification results, suggesting that AI applications in HAR are coming closer to 100% accuracy and that at present, SKS-transformer is surpassing other published models. The same holds for IoT IMU collected datasets, where the SKS-Transformer model outperformed the other models by 2.86% and 0.46%. Although modular system design with replaceable components allows for the classifier model to be replaced, a steady performance of the SKS-transformer suggests its potential for extending the contexts of external validity, hence suggesting the generalizability and potential transferability of this study and experimental findings to broader populations, experimental settings, and contexts beyond the specific research environments covered in this study.

Another limitation of the collected golf dataset is a relatively small but consistent improvement of SKS-Transformer (0.46%) in swing error detection over the best of 11 existing models, Ensem-HAR (38). Given that the SKS-Transformer model already achieves 100% accuracy on this dataset, this highlights the need to expand the scope of future data collection, highlighting potential for further applications of AI in sport coaching, and human motion modeling and analysis in general. In contrast, extensive evaluations on public benchmarks (UCI HAR, PAMAP2) and two real-world sensor datasets (self-collected HAR and golf swing error detection) demonstrate that the proposed SKS-Transformer consistently delivers strong performance and robust generalization under realistic noise and sensor placement variability, validating both the model architecture and the end-to-end sensing-inference pipeline.

### 4.2 Summary of design aspects, insights, and contributions

As part of the research rigor, ablation studies were intended to validate the contribution of each architectural component to overall performance. In summary:

- Gated fusion was found pivotal when activities are heterogeneous,
- Multi-scale and directional pathways provide complementary cues, and
- Light denoising stabilizes attention and improves feature coherence.

Inferred from the ablation studies, the key empirical insights are summarized as follows:

- **Learned fusion is pivotal on heterogeneous activities:** On the multi-activity benchmark, removing the gating/fusion module caused the largest degradation, indicating that a sample-dependent combination of multi-scale and directional cues is critical when activities vary widely in tempo and duration.
- **Multi-scale and directional cues are complementary:** Dropping either the selective-kernel branch or the axial (direction-aware) attention consistently hurt performance, showing that short, sharp motion motifs and orientation-sensitive dynamics encode distinct, non-substitutable information.
- **Attention is indispensable for fine-grained biomechanics:** In golf swing error detection, eliminating attention entirely led to the clearest decline, whereas removing any single pathway had smaller, similar effects, suggesting partial redundancy among submodules but a hard dependence on attention to resolve subtle kinematic differences.
- **Light preprocessing improves downstream learning:** Skipping low-pass/smoothing reduced performance even with strong attention, implying that small reductions in sensor noise make attention weights more stable and features more coherent.
- **End-to-end viability is confirmed:** High performance on the two sensor-collected datasets indicates that the observed accuracy stems from the combined effect of acquisition fidelity, labeling protocol, and modeling, i.e., the system works as a whole, not just the classifier in isolation.
- **Ceiling effects reveal next evaluation needs:** Near-ceiling results on the golf task imply strong class separability; future stress tests should emphasize domain shifts (sensor placement, speed/tempo changes, unseen users) to probe limits beyond clean separations.
- **Neural information processing workflow for IMU time series:** The experimentally validated modeling approach, comprised of multi-scale features + directional attention + learned fusion + light denoising, emerges as a general design pattern template for noisy IMU-based sequence recognition beyond HAR.

## 5 Conclusion and future work

In this paper, we introduced an end-to-end human activity recognition (HAR) system that couples a low-cost, custom-made IoT IMU-based sensor for wireless data streaming. The system is also coupled with the developed SKS-Transformer, which unifies scale-adaptive selective kernels, direction-aware axial attention, and learned gated fusion.

The performance of the modular system with SKS-Transformer was evaluated on two public datasets, UCI-HAR (97.01%) and PAMAP2 (98.0%), achieving new state-of-the-art performance (outperforming 11 previously published models by 0.3% and 0.09%, respectively). Additionally, the system was evaluated on two additional datasets captured using a custom-made JY61 ESP8266 IMU sensor. The system with SKS-Transformer consistently outperformed strong CNN–based and attention-based models, achieving 98.10% accuracy [compared to Ensem-HAR (38), which achieved 95.24%], hence providing evidence that the SKS-Transformer is extending the original applications of transformer-based AI models in text and video processing to HAR contexts. Additionally, the system achieved 100% accuracy in golf swing error detection, a fine-grained athletic activity, surpassing Ensem-HAR by 0.46%. From a research rigour perspective, the ablation study was useful to isolate the sources of SKS-Transformer performance gains, resulting in the following insights and discoveries: gated fusion is pivotal when activities are heterogeneous; multi-scale and directional pathways provide complementary cues; and light denoising stabilises attention and improves feature coherence. The results across the four datasets validate not only the architecture and SKS-Transformer, but also the entire HAR framework as an information-processing pipeline for real-life activities and associated data-collection conditions.

Future work will focus on advancing SKS-Transformer by addressing the three immediate investigation objectives:

**Efficient edge deployment:** We will compress SKS-Transformer via quantization-aware training and structured pruning, fuse operators, and co-design kernels with MCU constraints to preserve accuracy while meeting real-time latency and power budgets.

**Subject-independent generalization:** We will adopt a large-scale leave-one-subject-out evaluation and develop meta-learning objectives with few-shot, on-device personalization heads, enabling the model to rapidly adapt to a new user without overfitting or sharing raw data.

**Robustness to domain shift:** We will pursue self-supervised, continual adaptation from unlabeled streams to handle sensor hardware changes and placement drift, augment this with placement-aware normalization/calibration, and integrate calibrated uncertainty estimation to gate predictions, trigger recalibration, or fall back to a safer alternative in safety-critical settings.

As a primary objective for the benefit of global society, we also plan to make the JY61 ESP8266 IMU sensor open-source software and hardware, aiming to democratize consumer-grade technology and provide solutions for privacy-preserving human motion data streaming and processing.

## Data Availability

The raw data supporting the conclusions of this article will be made available by the authors, without undue reservation.

## Footnotes

^1^ https://archive.ics.uci.edu/dataset/240/human+activity+recognition+using+smartphones

^2^ https://archive.ics.uci.edu/dataset/231/pamap2+physical+activity+monitoring

^3^ https://doi.org/10.24432/C54S4K

^4^ https://doi.org/10.24432/C5NW2H
